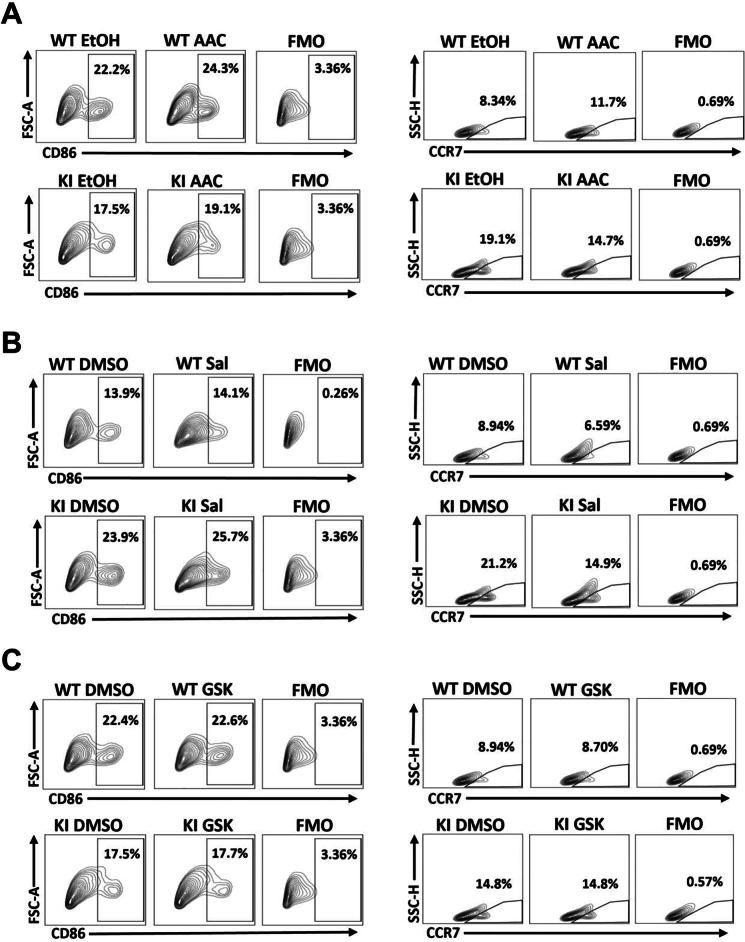# β2-Integrins regulate dendritic cells through nuclear deformation and activation of phospholipase A2 and DNA damage–inducible GADD34

**DOI:** 10.1016/j.jbc.2025.111093

**Published:** 2025-12-22

**Authors:** Riku Somermäki, Heidi Harjunpää, Yunhao Cui, Andrea Walander, Susanna C. Fagerholm

**Affiliations:** Molecular and Integrative Biosciences Research Programme, Faculty of Biological and Environmental Sciences, University of Helsinki, Helsinki, Finland

**Keywords:** dendritic cell, integrin, nuclear structure, endoplasmic reticulum stress, cell signaling

## Abstract

Dendritic cells (DCs) reside in tissues and are activated by danger or pathogen-associated signals, leading to expression of costimulatory markers and cytokines, downregulation of β2-integrin–mediated adhesion, migration to lymph nodes, and activation of T cells. Conversely, β2-integrins are known to restrict DC activation and migration, but signaling pathways involved in this process remain poorly understood. Here, we show that β2-integrins regulate podosome formation as well as nuclear morphology of bone marrow–derived DCs (BMDCs) on both stiff and soft surfaces. Analysis of published gene expression data revealed that loss of β2-integrin adhesion and nuclear deformation both upregulate DC activation markers and cytokines, including Ccr7, Cd86, and Il12b. Growth arrest and DNA damage–inducible protein (GADD34; Ppp1r15) of the unfolded protein responses was also upregulated in both datasets. Utilizing a cytosolic phospholipase A2 (cPLA2) inhibitor, we show that the nuclear shape sensor cPLA2 controls interleukin-12 production and CD86 expression of β2-integrin adhesion–deficient BMDCs. We further show that the GADD34 pathway is activated in adhesion-deficient BMDCs, as eukaryotic translation initiation factor 2A (EIF2α) phosphorylation is reduced in these cells, whilst cPLA2 inhibition rescues EIF2α phosphorylation. Furthermore, GADD34 inhibition led to decreased interleukin-12 production and CD86 expression in β2-integrin–deficient BMDCs, whilst inhibition of protein kinase R–like endoplasmic reticulum kinase (the canonical EIF2α kinase) had no effect. Together, our results show that loss of β2-integrin adhesion leads to nuclear deformation and activation of a cPLA2–GADD34 pathway in BMDCs, which at least partly controls their activated phenotype. Our results therefore reveal how cell adhesion and nuclear deformation are connected to mechanically control DC activation.

Integrins are αβ heterodimeric adhesion receptors, which can simultaneously bind to extracellular components in the cell’s microenvironment and the cytoskeleton and signaling proteins inside the cell. Integrins relay mechanical signals to the nucleus through the actin cytoskeleton, which is tethered to the nuclear lamina *via* the linker of nucleoskeleton and cytoskeleton complexes to regulate transcription and chromatin state (1). Moreover, the cell can adapt integrin ligand binding by conformational changes of its extracellular domain (2). β2-Integrins are a subgroup of integrins expressed exclusively in leukocytes (3). β2-Integrins share a common β2 chain (CD18) but can have four different α-chains, with different ligand specificities and functions (CD11a/CD18, αLβ2, LFA-1; CD11b/CD18, αMβ2, Mac-1, CR3; CD11c/CD18, αXβ2, p150.95, CR4; and CD11d/CD18, αDβ2). The β2-chain cytoplasmic tail interacts with the cytoplasmic regulator kindlin-3 through its distal NXXF (where X is any amino acid) motif (4), and TTT motif, and this interaction is crucial for leukocyte adhesion (5). β2-Integrin adhesion is essential for leukocyte trafficking, immune synapse formation, cytokine responses, and cellular reprogramming (3).

During infection, dendritic cells (DCs) take up antigens and are activated (maturation or reprogramming) in response to pathogen-associated and danger signals, leading to C–C chemokine receptor type 7 (CCR7)–guided migration into lymph nodes for initiating adaptative immune responses (6). We have shown that loss of β2-integrin adhesion (through TTT/AAA mutation of the kindlin-3 binding motif in the β2-tail) reprograms bone marrow–derived DCs (BMDCs) into an activated, migratory DC-like subtype seen as, for example, increased CCR7 expression, cytokine interleukin 12 (IL-12) production, costimulatory marker expression, *in vitro* migration, and *in vivo* tumor rejection (7, 8). Integrin dysfunction abolishes filamentous actin (F-actin)–driven Ras homolog family member A (RhoA) activation, preventing myocardin-related transcription factor A nuclear localization–driven gene expression (9). Moreover, loss of integrin adhesion in DCs leads to reduced nuclear scaffold lamin A/C levels, increased histone methylation (H3K4me3 and H3K27me3), transcriptional changes, and an altered metabolic landscape (8, 10). β2-Integrin adhesion thus controls DC reprogramming, which we have termed “mechanical immune memory.” Loss of adhesion may be a “danger signal” leading to activation of DCs. However, the signaling pathways and molecular mechanisms by which β2-integrins regulate DC reprogramming are still poorly understood.

As the DC was activated, the migratory phenotype has recently been shown to be regulated through nuclear deformation–induced cytosolic phospholipase A2 (cPLA2) activation (11). We here turned to investigate the role of β2-integrin–mediated adhesion in regulating this pathway in DCs. We show that loss of β2-integrin adhesion led to a fundamentally altered actin cytoskeleton and nuclear deformation in BMDCs, which prompted us to study the role of cPLA2 in integrin-regulated DC reprogramming. Indeed, we show that cPLA2 activity controls the DC activation markers of β2-integrin adhesion–deficient BMDCs. Interestingly, our RNA-Seq data analysis identified both nuclear deformation and β2-integrin adhesion to regulate the eukaryotic translation initiation factor α phosphatase Ppp1r15 (encoding growth arrest and DNA damage–inducible protein [GADD34]) level in DCs. We show that GADD34 partly regulates the DC activation markers of β2-integrin–deficient DCs. Our results indicate that β2-integrin adhesion restricts DC activation at least in part *via* nuclear deformation–induced cPLA2/GADD34 activity, shedding light on the mechanisms by which β2-integrins control DC reprogramming.

## Results

### Loss of β2-integrin adhesion results in an altered actin cytoskeleton and nuclear deformation of BMDCs

**Figure 1:**
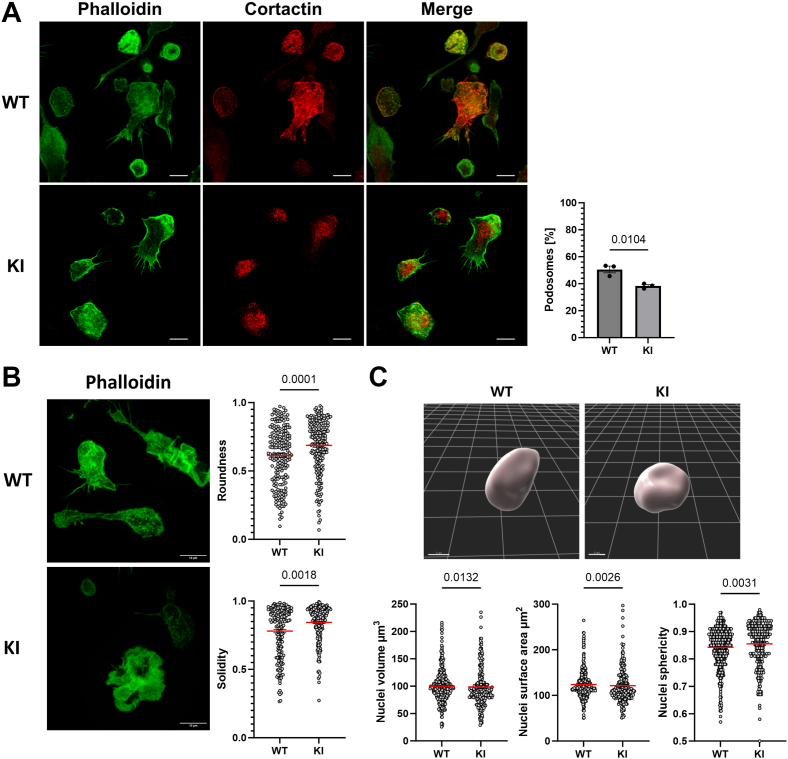
**β2-Integrin adhesion controls actin cytoskeleton dynamics and nuclear morphology.***A*, representative figures of WT- and β2-integrin–deficient (KI) DCs on a glass surface. Podosomes were identified by colocalization of cortactin and phalloidin-positive dot-like structures on the glass surface. Three biological replicates, WT n = 346, KI n = 295 cells analyzed, the scale bar represents 10 μm. *B*, representative figure of WT- and KI DC actin structure/cell shape. Two biological replicates, WT n = 206, KI n = 216 cells, were analyzed; the scale bar represents 10 μm. *C*, representative 3D rendered nuclei of WT DC and KI DC, the grid represents 5 μm. Three biological replicates, WT n = 357, KI n = 255 nuclei analyzed. Unpaired *t* test was used to assess the *p* value of podosomes, whereas Mann–Whitney *U* tests were used for the rest of the experiments. Data are represented as mean and ±SEM. KI DC, knock-in bone marrow–derived dendritic cell.

We investigated the actin cytoskeleton, adhesion, and nuclear phenotype of integrin TTT/AAA β2-integrin knock-in BMDCs (from here on called KI DCs), which we have previously shown to display an activated phenotype (7, 8). We stained the actin cytoskeleton and the podosome marker cortactin in WT and KI cells and confirmed here that KI DCs have reduced podosome formation (actin- and cortactin-rich dot-like adhesive structures) when placed on a hard surface, for example, glass (Fig. 1*A*) (12). In addition, the cells displayed a loss of filopodia, leading to increased roundness and solidity of the cells (Fig. 1*B*). In lipopolysaccharide-stimulated DCs, loss of adhesion has recently been postulated to contribute to nuclear deformation (13). We used confocal microscopy to investigate whether loss of β2-integrin adhesion induces nuclear deformation in BMDCs. Indeed, the KI DCs have significantly smaller and more spherical nuclei (both 3D volume and surface area), reflecting a more compact, rounded morphology than nuclei of WT DCs (Fig. 1*C*). Together, these results confirm that loss of cell adhesion (because of integrin mutation) leads to significant alterations in morphology of BMDCs, including nuclear deformation.

### Substrate stiffness controls DC nuclear morphology and IL-12 production

**Figure 2:**
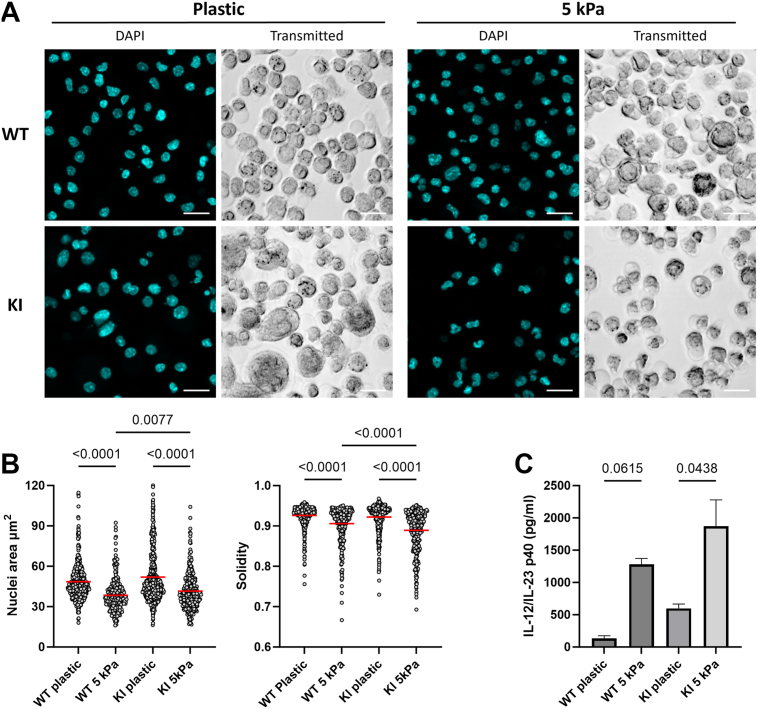
**Substrate stiffness controls BMDC nuclear morphology and cytokine production.***A*, representative figures of WT- and β2-integrin–deficient (KI) DCs placed on plastic, or 5 kPa Young’s modulus hydrogels. The scale bars represent 20 μm. *B*, nuclear area and solidity of WT- and KI DCs on plastic and 5 kPa substrate. Two biological replicates WT 5 kPa n = 338, plastic n = 510, KI plastic n = 531, 5 kPa n = 337 nuclei analyzed. *C*, WT- and KI DCs interleukin-12 production on plastic *versus* hydrogel substrate *via* ELISA n = 2. Kruskal–Wallis test followed by Dunn’s post hoc test was used to acquire *p* value for nuclear studies, whereas ANOVA followed by Tukey’s post hoc test was used for ELISA. Data are represented as mean and ±SEM. BMDC, bone marrow–derived dendritic cell; KI DC, knock-in bone marrow–derived dendritic cell.

A softer substrate is known to substantially downregulate integrin-mediated adhesion of cells (14, 15, 16), thereby mimicking the effect of the integrin KI mutation. To further investigate the effect of integrin-mediated adhesion on nuclear size and morphology, we placed WT- and KI DCs on a softer substrate, for example, a 5 kPa Young’s modulus hydrogel (which more closely mimics the natural tissue environment of these cells). We found that on this softer substrate, WT cells displayed a much smaller nucleus than on a stiffer substrate, for example, plastic, showing that reduced adhesion of DCs on softer substrates also regulates nuclear size (Fig. 2, *A* and *B*). KI DC nuclei on the softer substrate were not significantly smaller than those of WT cells, but the nuclei showed a substantially aberrant morphology, akin to that of lamin A/C KO cells (17), with the nuclei losing their normal, rounded morphology (Fig. 2, *A* and *B*). We further investigated WT- and KI DC IL-12 production (a marker of BMDC activation) on soft and stiff substrates. We found that WT DCs placed on softer substrates produced significantly more IL-12 than on plastic, and also KI cells slightly upregulated IL-12 production on softer substrates than on stiffer substrates (Fig. 2*C*). All in all, the results show that integrin-mediated adhesion to the substrate regulates DC nuclear size and morphology as well as DC activation status.

### Β2-Integrins and nuclear deformation regulate similar gene targets in BMDCs

**Figure 3:**
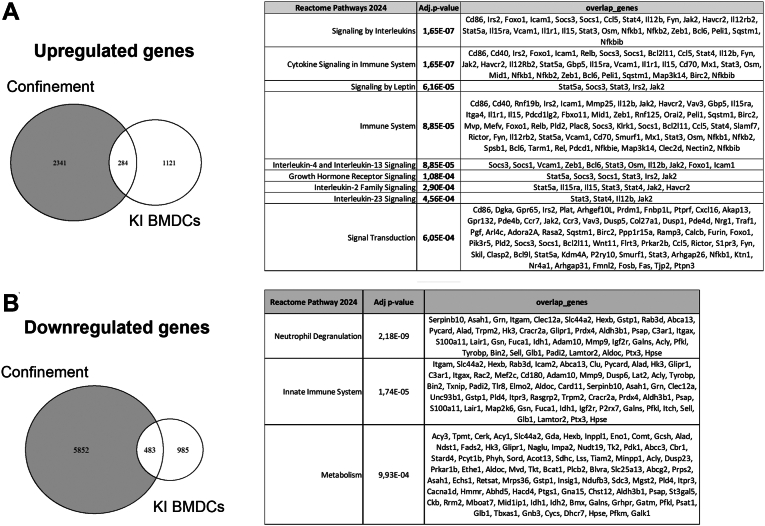
**Nuclear confinement and β2-integrin adhesion regulate shared pathways.***A* and *B*, nuclear confinement updownregulated genes of BMDCs (11) overlap with β2-integrin adhesion–deficient (KI) BMDCs (8). BMDC, bone marrow–derived dendritic cell; KI DC, knock-in bone marrow–derived dendritic cell.

Recently, nuclear deformation has been shown to regulate the activated, migratory DC phenotype through controlling transcription (11). As we have previously shown that β2-integrins also control DC activation and migration, we here investigated a potential connection between integrin adhesion, nuclear deformation, and gene transcription by investigating previously published gene expression signatures of nuclearly deformed BMDCs and KI DCs. Of the 284 shared upregulated genes (Table S1), Reactome pathway analysis highlighted interleukin, cytokine, and immune cell signaling to be both nuclear deformation and β2-integrin adhesion controlled (Fig. 3*A*). More precisely, genes, such as Ccr7, Stat5a, Cd40, Cd86, Il12b, and Nfkb1, were upregulated under both these conditions, indicating a potential similar mechanism between these different types of stimuli, which both regulate the activated DC phenotype.

We have recently shown that beta2-integrins regulate metabolic reprogramming and activation of DCs at least partly through the transcription factor Ikaros (8, 10). Interestingly, in the shared downregulated gene group, Reactome pathway analysis of the 483 shared genes shows metabolism genes to be downregulated both by loss of adhesion and by nuclear squeezing (Fig. 3*B*). These metabolism genes included glycolytic genes such as Ikaros—targets Eno1 and Pfkl (18), which we have shown to be downregulated in beta2-integrin KI cells (10). Together, our omics approach suggests that both nuclear deformation and β2-integrin adhesion control activation and metabolic reprogramming of BMDCs.

### The lipid metabolism enzyme cPLA2 regulates the KI DC phenotype

**Figure 4:**
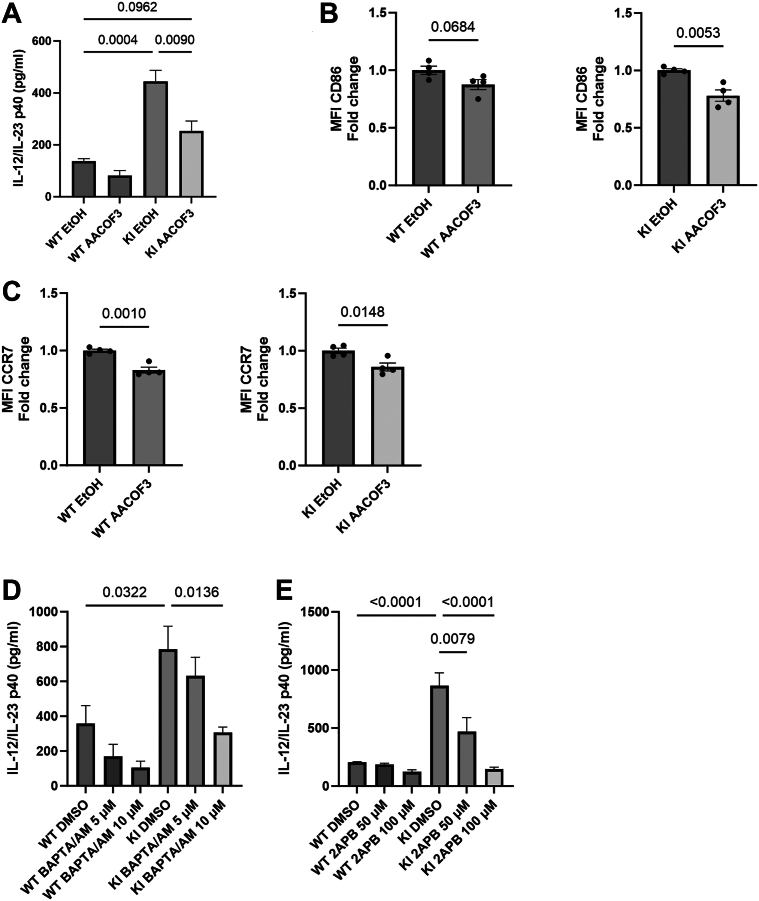
**cPLA2 controls β2-integrin adhesion–deficient (KI) DCs phenotype.***A*, cPLA2 inhibitor (AACOCF3) treated WT- and KI DC interleukin 2 (IL-12) production *via* ELISA (n = 3). *B* and *C*, AACOCF3-treated WT- and KI DCs CD86 and CCR7 expression assessed by flow cytometry (result pooled from two experiments, n = 4). *D*, calcium chelator BAPTA/AM–treated WT- and KI DC IL-12 production *via* ELISA (n = 4). *E*, IP_3_ receptor/store-operated Ca^2+^ inhibitor 2-APB treated WT- and KI DC IL-12 production *via* ELISA (n = 4). ANOVA followed by Tukey’s post hoc test was used for ELISA results to acquire *p* values, whereas unpaired *t* tests were used for flow cytometry results. Data are represented as mean ± SEM. CCR7, C–C chemokine receptor type 7; cPLA2, cytosolic phospholipase A2; KI DC, knock-in bone marrow–derived dendritic cell.

**Supplemental Figure 1:**
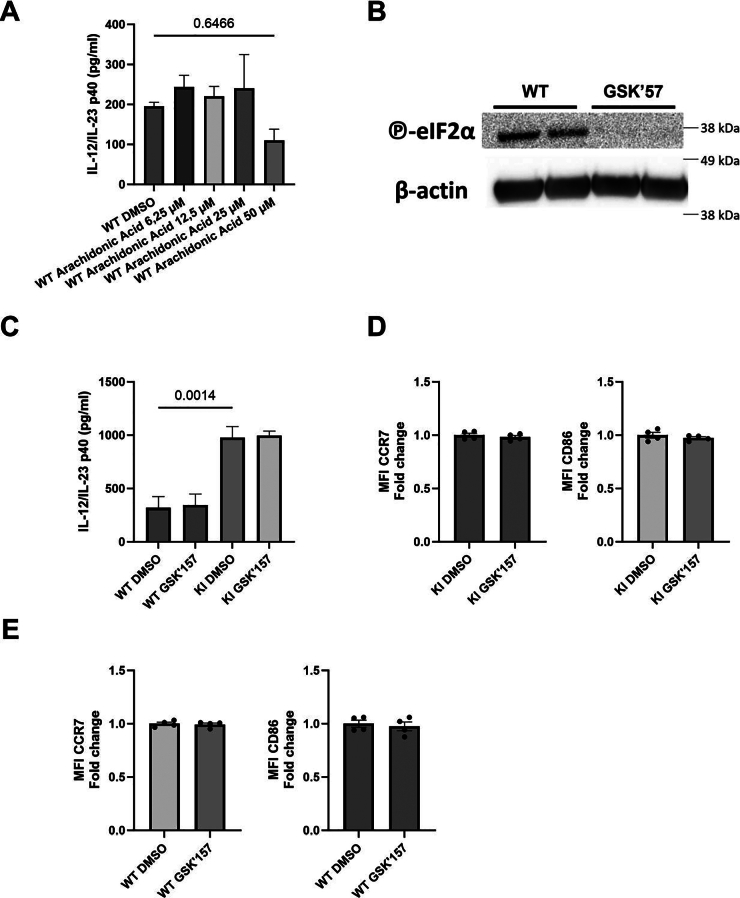


Recently, the lipid metabolism enzyme cPLA2 was shown to control DC transcription, migration, and CCR7 expression during nuclear squeezing (11). As these processes are also controlled by β2-integrin adhesion (7), and as β2-integrins also regulate nuclear morphology of BMDCs (Fig. 1*C*), we utilized a cPLA2 inhibitor (AACOCF3) to assess whether cPLA2 activity is involved in regulating the KI DC phenotype (Figs. 4 and S2*A*). Interestingly, cPLA2 inhibition led to significantly reduced IL-12 and CD86 expression in KI DCs but not in WT cells (Fig 4, *A* and *B*). In contrast, cPLA2 inhibition reduced CCR7 expression both in WT and KI DCs (Fig. 4*C*). Furthermore, Ca^2+^ signaling, which has previously been shown to be critical for cPLA2 activation upon nuclear deformation (19, 20), was also found to regulate KI DC IL-12 levels, using BAPTA/AM (which chelates intracellular calcium) and 2APB (which blocks Ca^2+^ release from intracellular stores) (Fig. 4, *E* and *F*). These experiments provide further evidence in support of a role for Ca^2+^–cPLA2 in integrin-regulated IL-12 production in DCs. In contrast, arachidonic acid, the lipid product of the cPLA2 enzyme, when added to the cell media, was not able to activate WT DCs (Fig. S1*A*). This may be due to altered localization of this metabolite in the WT cells when administered extracellularly (not localized specifically at the nuclear/endoplasmic reticulum [ER] membrane). Together, these results suggest that integrin-regulated nuclear deformation and cPLA2 activation at least partly control the β2-integrin–restricted DC activation.

### cPLA2 and β2-integrin adhesion control phospho-EIF2α levels in BMDCs

**Figure 5:**
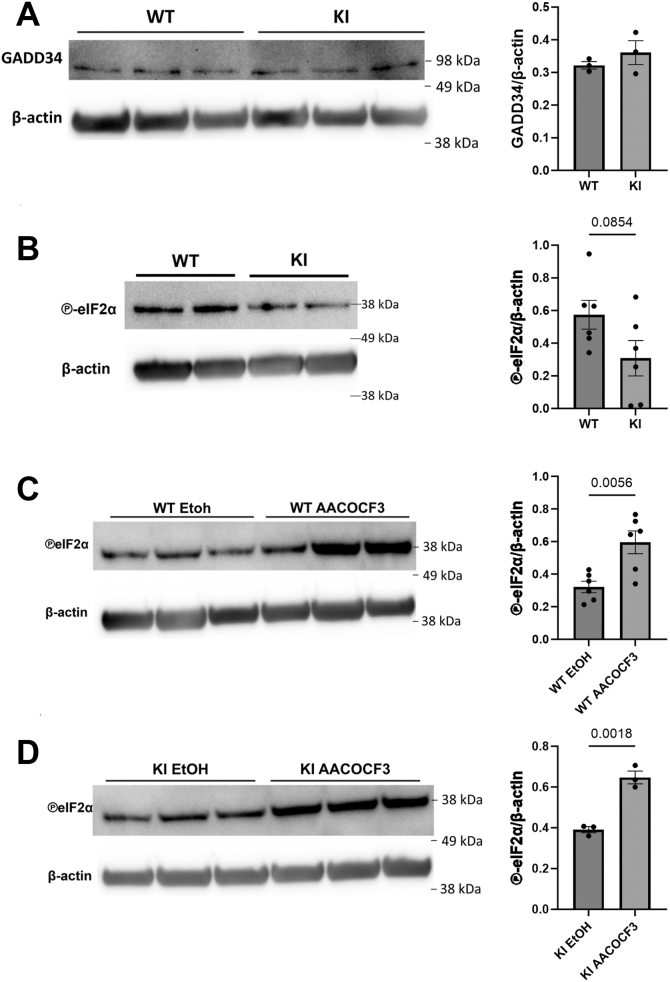
**Crosstalk between cPLA2 and GADD34 pathways in BMDCs.***A*, GADD34 protein level measured by Western blot (n = 3). *B*, representative figure of phospho-EIF2α level measured by Western blot (n = 6). *C*, representative figure of phospho-EIF2α level measured by Western blot in cPLA2-inhibited (AACOCF3) WT BMDCs (n = 6), and (*D*) KI BMDCs (n = 3). Unpaired t tests were used to acquire *p* values. Data are represented as mean ± SEM. BMDC, bone marrow–derived dendritic cell; cPLA2, cytosolic phospholipase A2; EIF2α, eukaryotic translation initiation factor 2A; GADD34, growth arrest and DNA damage–inducible protein.

We analyzed the RNA-Seq data (Fig. 3*A*, Table S1) for potential signaling pathways regulated by both β2-integrins and cPLA2 (8, 11). Interestingly, Reactome pathway analysis of gene expression data also highlighted “signal transduction” with potential crosstalk targets for cPLA2 in BMDCs, such as the EIF2a phosphatase Ppp1r15a (encoding GADD34). BMDCs employ the phosphatase GADD34 of the unfolded protein responses (*via* EIF2α Ser51 phosphorylation (21)) to maintain protein synthesis and adjust cytokine production during stimulation, while also regulating BMDC migration and activation (22, 23). Although Ppp1r15 mRNA levels were upregulated in KI DCs, we did not detect a change in GADD34 protein levels (Fig. 5*A*). However, KI DCs displayed a trend toward lower phospho-EIF2α (Fig. 5*B*) levels compared with WT cells, implicating increased EIF2α phosphatase activity. Interestingly, cPLA2 inhibition increased phospho-EIF2α levels in both WT and KI cells (Fig. 5, *C* and *D*), suggesting crosstalk between the GADD34- and cPLA2 pathways in BMDCs.

### The GADD34 phosphatase controls cytokine production and surface marker expression in BMDCs

**Figure 6:**
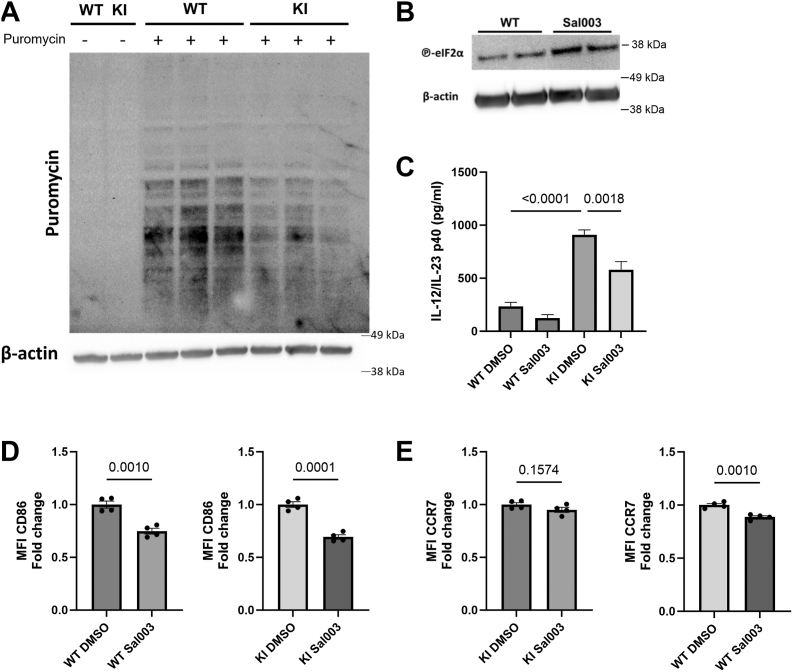
**GADD34 partly controls the maturation of β2-integrin adhesion–deficient (KI) DCs.***A*, total translation assessed by puromycin incorporation followed by Western blotting in WT- and KI BMDCs (n = 3). *B*, GADD34 inhibitor (Sal003) was validated to control phospho-EIF2α levels of BMDCs by Western blotting, representative figure (n = 4). *C*, interleukin-12 production by ELISA in GADD34-inhibited WT and KI cells (n = 5). *D* and *E*, flow cytometry on costimulatory marker CD86 and activation marker CCR7 expression in GADD34-inhibited WT and KI cells (result pooled from two experiments, n = 4). ANOVA followed by Tukey’s post hoc test was used to acquire *p* value for the ELISA experiment, whereas unpaired *t* tests were used for the flow cytometry experiments. Data are represented as mean ± SEM. CCR7, C–C chemokine receptor type 7; EIF2α, eukaryotic translation initiation factor 2A; GADD34, growth arrest and DNA damage–inducible protein; KI DC, knock-in bone marrow–derived dendritic cell.

As we found a trend for reduced phospho-EIF2α levels that were also regulated by cPLA2 activity in KI DCs, next, we turned to explore the putative roles for phospho-EIF2α (Ser51)–mediated ER stress responses in regulating the phenotype of KI DCs. ER stress is known to control translation in stressed cells (24), and we noted reduced translation in KI DCs (Fig. 6*A*). We then validated the functionality of the GADD34 inhibitor Sal003 in BMDCs, showing that pretreatment of cells with Sal003 led to increased BMDC phospho-EIF2α levels (Fig. 6*B*). We found that GADD34 inhibition by Sal003 pretreatment significantly reduced IL-12 production in KI DCs but not significantly in WT BMDCs (Fig. 6*C*). However, both WT and KI cells treated with GADD34 inhibitor exhibit reduced CD86 expression, whilst inhibition of GADD34 only reduced CCR7 expression in WT BMDCs (Figs. 6, *D*, *E* and S2*B*).

Interestingly, ablating phospho-EIF2α by inhibiting its kinase PERK (protein kinase R–like ER kinase) (25) (with GSK2656157; Fig. S1*B*) did neither alter cytokine production nor control surface marker expression in any of our experiments (Fig. S1, *C*–*E*). Together, these results indicate β2-integrins control IL-12 expression through GADD34 activity but not through regulating phospho-EIF2α levels, for example, the unfolded protein response. In contrast, costimulatory marker expression is regulated by GADD34 both in WT and KI cells, indicating that this process may not be dependent on integrin but is dependent on the GADD34 phosphatase.

## Discussion

Many myeloid cells, such as macrophages and DCs, reside mainly in tissues where they are continuously exposed to extracellular mechanical cues. Allosteric adhesion receptors called integrins are essential mechanosensory links between the cell and its environment (16, 26). We have shown that the leukocyte-specific β2-integrin–kindlin-3 interaction controls DC adhesion, signaling pathways (*e.g.*, RhoA, Syk, and many downstream pathways), transcriptional landscape (*e.g.*, H3K4me3, H3K27me3, TFs Ikaros, NF-κB), and metabolic programming (*e.g.*, Hk2, Pfkl, GLUT1, reactive oxygen species, and ATP production), which ultimately restricts DC activation (*e.g.*, *in vivo* tumor suppression, CCR7, CD86, IL-12) (5, 7, 8, 9, 10). We call this process “mechanical immune memory,” as integrin-mediated interactions with the cell’s mechanical microenvironment support the immature state of DCs and therefore homeostasis, whilst loss of adhesion may constitute a danger signal, inducing DC activation.

β2-Integrin’s intracellular tail interacts with adapter proteins, talin and kindlin-3, to connect it to the actin cytoskeleton (27, 28, 29). Here, we show that DCs with β2-integrin deficiency in kindlin-3 binding (KI DCs) exhibited altered F-actin dynamics with reduced filopodia and podosome formation. We have shown that DCs can be activated by growing them in suspension or by disrupting the F-actin cytoskeleton with cytochalasin D treatment (7, 8). F-actin dynamics are involved in nuclear morphology and DC reprogramming as for example, nuclear deforming by F-actin depolymerizing enzyme, cofilin-1 (through atypical Ser41 phosphorylation), aids DC migration through the extracellular matrix (13). Furthermore, it is known that integrins link directly to the cell nucleus *via* actin and the linker of nucleoskeleton and cytoskeleton–lamin complex (30). We show here that β2-integrins regulate DC nuclear size on hard surfaces (glass). Furthermore, we show that placing WT DCs on a softer substrate (*e.g*., 5 kPa Young’s modulus hydrogel) leads to significantly altered nuclear morphology (smaller nucleus), whilst the nuclei of KI cells displayed further nuclear dysmorphia and were floppier on the soft substrate. We have previously shown that β2-integrins regulate lamin A/C levels in cells (8). We speculate that loss of β2-integrin mechanosignaling reduces lamin A/C levels, which may lead to a softer nuclear envelope, aiding nuclear deformation (31, 32). Thus, our previously published results, in conjunction with results presented here, suggest that β2-integrins control nuclear dynamics through reducing lamin A/C levels, which may lead to a softer nucleus, which is more susceptible to detecting mechanical changes.

Mechanically squeezing DC nuclei has previously been shown to induce CCR7-guided migration to lymph nodes *via* nuclear shape sensing–lipid metabolism enzyme cPLA2 activity (11). We found that both nuclear squeeze stress and loss of β2-integrin adhesion control gene expression programs associated with DC activation (*e.g.*, Ccr7, Il-12, Cd86, etc). In line with the altered nuclear shape and size in integrin-deficient cells, we confirmed that cPLA2 regulates costimulatory marker expression and cytokine production in integrin-deficient KI DCs. Calcium controls cPLA2 activity and translocation to the nuclear membrane during nuclear deformation (19), where cPLA2 activity produces arachidonic acid *in situ* (20). Blocking calcium from entering the cell or blocking calcium release from intracellular storages reduced KI DC cytokine production, further supporting a role of this pathway in regulating the KI DC–activated phenotype. However, extracellular addition of arachidonic acid to WT DCs did not affect cytokine production, suggesting perhaps a downstream lipid metabolite instead is important, or that the perinuclear ER–nuclear localization of cPLA2 enzymatic activity is essential for the regulation of DC activation.

**Figure 7:**
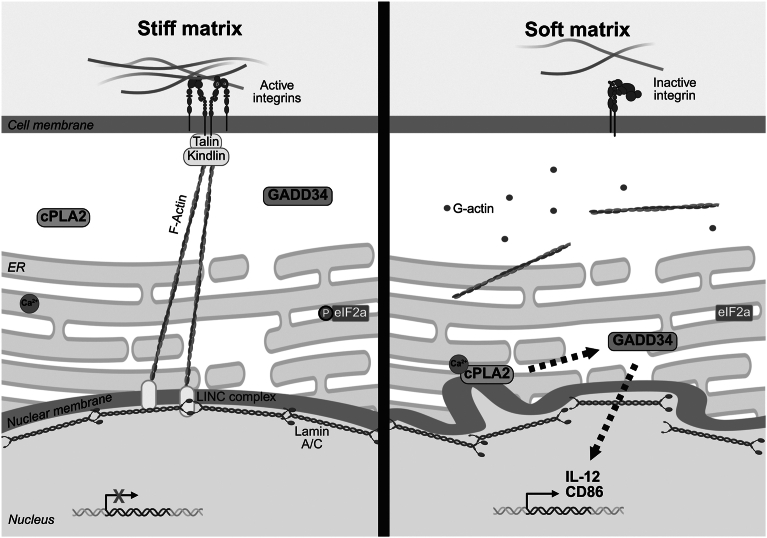
**Loss of β2-integrin adhesion leads to nuclear dysmorphia and activation of a cPLA2–GADD34 pathway in DCs.***Left,* on a stiff matrix, integrins are active and connected to the actin cytoskeleton *via* adapters such as talin and kindlin. F-actin connects integrins to the linker of nucleoskeleton and cytoskeleton (LINC) complex and nuclear lamina (lamin A/C) to suppress gene expression. *Right,* on a soft matrix, integrins are inactive, and the cytoskeletal structure is lost, leading to nuclear dysmorphia and Ca^2+^ controlled cPLA2 activation. cPLA2 (directly or indirectly) interacts with GADD34, which ultimately promotes interleukin-12 and CD86 expression in DCs. cPLA2, cytosolic phospholipase A2; DC, dendritic cell; F-actin, filamentous actin; GADD34, growth arrest and DNA damage–inducible protein.

Our RNA-Seq data analysis identified upregulation of phosphatase GADD34 of the unfolded protein response as both a nuclear deformation and β2-integrin adhesion–controlled target. Interestingly, cPLA2 inhibition led to increased phosphorylation of EIF2α, the direct target of GADD34 (21), suggesting crosstalk between GADD34 and cPLA2 pathways. Finally, we show that GADD34 partly controls DC activation in β2-integrin–deficient DCs, for example, CD86 expression and cytokine production. Interestingly, ablating phospho-EIF2α by inhibiting its kinase PERK did not influence the cytokine production or maturation marker expression in any of our experiments. In contrast, in lipopolysaccharide-stimulated DCs, PERK activity promotes migration and cytokine production (22), whereas DCs infected with respiratory syncytial virus use PERK to restrict IL-12a mRNA level, and ATP and reactive oxygen species expression (33), supporting the idea that the PERK axis modulates DC reprogramming in response to different stimuli. Thus, the results here suggest that the β2-integrin–nuclear deformation–cPLA2/GADD34 pathway controls activated DCs (Fig. 7), potentially through effects of GADD34 on transcription (23). It is noteworthy that both cPLA2 and β2-integrins have been reported to regulate NF- κB signaling and NF- κB-regulated gene expression in DCs (8, 11). However, a limitation of this study is that the proposed pathway is supported only by the use of single inhibitors and biochemical analyses. Future studies incorporating genetic approaches will be necessary to confirm whether GADD34 regulates NF- κB signaling downstream of cPLA2 to regulate gene expression and DC activation.

It is apparent that β2-integrins are central regulators of DC activation. Consequently, comprehension of their downstream signaling processes is key for understanding the signaling events and mechanisms involved in mechanical immune memory, where changes in mechanical forces detected by immune cells lead to altered immune cell function and immune responses.

## Experimental procedures

### Mice and BM collection

Integrin TTT/AAA β2-integrin KI mice have been previously described (5). C57BL/6N WT littermates were purchased from Charles River and were used as controls for β2-integrin KI mice. Experiments were performed using male and female mice between the ages 8 and 39 weeks. Animals were housed under conventional conditions in groups of up to five animals per cage with free access to food and water. BM was collected from euthanized animals and used for BMDC cultures immediately. All the experiments were performed according to the Finnish Act on the Protection of Animals Used for Scientific or Educational Purposes (497/2013) and to the Directive 2010/63/EU of the European Parliament and of the Council of September 22, 2010 on the protection of animals used for scientific purposes and approved by the Finnish National Animal Experiment Board (Hankelupalautakunta—ELLA).

### BMDC culture

BMDCs were generated by culturing BM cells for 9 to 10 days in DC media: 10 ng/ml granulocyte–macrophage colony-stimulating factor (Peprotech; catalog no.: AF-315-03) in RPMI (Lonza; catalog no.: 12-167F/EuroClone, catalog no.: ECB9006L) supplemented with 10% fetal calf serum (Gibco, catalog no.: 10500-064), 100 U/ml penicillin–streptomycin (penicillin, Orion, catalog no.: 465161; streptomycin, Thermo Fisher Scientific, catalog no.: D7253-100 g), and 2 mM l-glutamine (Thermo Fisher Scientific, catalog no.: BP379-100), at 37 °C in a humidified atmosphere of 5% CO_2_. Media were added/changed on days 3, 6, and 8. On days 8 and 9, in some experiments specified in figure legends, the cells were treated with 10 μM Sal003 (8 h for Western blotting [WB] experiments, overnight for ELISA experiments, Millipore, catalog no.: 324896-5mg), 1 μM GSK2656157 (overnight, Millipore, catalog no.: 5.04651.0001), 25 μM AACOCF3 (overnight, Tocris, catalog no.: 1462), 5 or 10 μM BAPTA/AM (overnight, Merck, catalog no.: 196419-25MG), 50 or 100 μM 2-APB (overnight, Merck, catalog no.: 100065-100MG), and 6.25 to 50 μM arachidonic acid (overnight, Sigma–Aldrich, catalog no.: 10931-250MG).

In some experiments, cells were plated onto 5 kPa Young’s modulus hydrogels (made according to the manufacturer’s protocol; Gelomics, LunaGel Ultrapure GelMA high stiffness, catalog no.: SKU0018) on days 8 and 9 of the BMDC culture. Briefly, 5 kPa LunaGels were prepared by mixing photoinitiator in PBS and 37 °C prewarmed GelMA. The mixture was then transferred to an empty 48-well plate (CellStar, catalog no.: 677180) and crosslinked using LunaCrosslinker (Gelomics) for 32 s to achieve 5 kPa Young’s modulus stiffness. One million BMDCs were then seeded on the gels and incubated at 37 °C 5% CO_2_ overnight.

### Confocal microscopy

BMDCs (0.75 × 10^6^) were seeded on glass coverslips at 37 °C in a humidified atmosphere of 5% CO_2_ for 4 h. The cells were fixed with 4% paraformaldehyde in PBS for 10 min. Cells were permeabilized using 0.2% Triton X in PBS for 5 min, blocked for 60 min with 0.1% Triton X, 3% bovine serum albumin (BSA) in PBS, and incubated with 16.7 μg/ml phalloidin–FITC (Sigma–Aldrich, catalog no.: P5282-1MG). In some experiments, after washing, cells were incubated with rabbit anti-Cortactin (H222) antibody (Cell Signaling, catalog no.: 3503S) and donkey anti-rabbit IgG (H + L) highly crossadsorbed secondary antibody, Alexa Fluor 594 (Invitrogen, catalog no.: A21207). Nuclei were stained with 4’,6-diamidino-2-phenylindole (DAPI) (Sigma, catalog no.: D9542), and coverslips were mounted with ProLong Glass antifade mountant (Invitrogen, catalog no.: P36982). Confocal microscopy images were acquired using an Olympus FV4000 confocal microscope numerical aperture of 1.42 60× objective, with 594 nm, 488 nm, and 405 nm lasers (3.5%, 0.1%, and 3.5% laser transmissivity, respectively). Z-stacks were taken with the following parameters: zoom: 1.00×; z-distance 0.35 μm (number of steps was manually selected to cover the whole phalloidin–FITC signal), format 800 × 800.

The figures were analyzed empirically for podosomes. Cell-containing podosomes were identified *via* colocalization of cortactin–Alexa Fluor 594 and Phalloidin–FITC dot-like structures in the glass-attaching surface.

The roundness (roundness = 4 × area/[π × major axis^2^], ranges from 0 to 1, with one indicating a perfect circle) and solidity (solidity = area/convex area, ranges from 0 to 1, with one indicating no concave defects) were quantified by hand-drawing outline of phalloidin–FITC signal of projected cells as region of interest (ROI) and measuring ROIs using ImageJ Fiji.

The 3D renders were acquired from z-stack figures of the DAPI channel using Imaris (Oxford Instruments). In short, we created a threshold intensity, which would define the boundary of the nuclei, and Imaris would then produce the 3D rendering of the nuclei. A representative subset of 3D renders was examined to confirm correspondence with the DAPI signal. The 3D rendered nuclei were analyzed for their volume, surface area, and sphericity (sphericity = πˆ(1/3) × (6 × volume)ˆ(2/3)/surface area, which ranges from 0 to 1, with one indicating a perfect sphere).

### Widefield microscopy

For analysis of nuclear area on hydrogels, BMDCs seeded on hydrogels (hydrogel thickness approximately 0.85 mm) for overnight and were fixed with 4% paraformaldehyde in PBS for 10 min. The cells were then permeabilized and blocked for 30 min using 0.1% Triton X, 3% BSA in PBS, and nuclei were stained with DAPI. The images were acquired in widefield mode on an Olympus FV4000 confocal microscope using a numerical aperture 0.4 10× objective with 405 nm lasers (9.9% laser transmissivity) and transmitted light with zoom 5.04× in 1024 × 1024 format. ROIs were acquired by thresholding the DAPI channel and using ImageJ Fiji’s Analyze Particles tool. Area and solidity of the ROIs were then measured.

All microscopy data analyses were done using GraphPad Prism (GraphPad Software, Inc).

### RNA-Seq data analysis

TTT/AAA beta2-integrin mice RNA-Seq data analysis has been described previously (8). Nuclear deformation–normalized RNA-Seq data were downloaded from the Gene Expression Omnibus database (GSE207653; (11)) and analyzed using the R Bioconductor Limma Voom package with adjusted *p* value <0.05 and log fold change ±0.3 set as cutoffs. A Venn diagram and the list of shared upregulated genes were acquired using RStudio (Posit PBC). Pathway enrichment analysis of shared upregulated and downregulated genes was performed using Enrichr (https://maayanlab.cloud/Enrichr/).

### Flow cytometry

The following conjugated antibodies were used for flow cytometric analysis of BM-DCs (company, catalog number, and clones are given in parentheses): CCR7-PE (BioLegend, catalog no.: 120105, clone 4B12), CD86-FITC (catalog no.: 11-0862-85, BD Biosciences, clone GL1). Fc-receptor block (BD Pharmingen, catalog no.: 553142, clone 2.4G2) was used, and unstained and fluorescence minus one controls were included in all experiments. Propidium iodide (Sigma–Aldrich, catalog no.: P4864) was used to detect dead cells. Acquisition was performed on an LSR Fortessa flow cytometer (Becton Dickinson), and data were analyzed using FlowJo software (Tree Star).

### ELISA

The cytokine levels were assessed with mouse IL-12/IL-23 p40 Allele-specific DuoSet ELISA (R&D, catalog no.: DY499) kit according to the manufacturer’s protocols. Briefly, a 96-well MaxiSorp microplate (Thermo Fisher Scientific, catalog no.: 442404) was first coated with capture antibody overnight. The next day, the wells were washed and blocked with 1% BSA (Biowest, catalog no.: P6154) in PBS (Lonza, catalog no.: 17-516F) for a minimum of 1 h. The plates were washed, and standards and samples were added. Following 2 h incubation, the plate was washed, and the detection antibody was added to the wells. After 2 h incubation, the plate was washed, and streptavidin–horseradish peroxidase was added for 20 min. After washing, the substrate solution (R&D, catalog no.: DY999) was added in the wells. Following visible color development (or latest after 20 min), sulfuric acid (Acros Organics, catalog no.: 124645001; diluted 1:4 in Milli-Q water) was added to stop the reaction. The absorbance was immediately determined at 450 nm, and the background at 540 nm wavelength using the Multiskan GO spectrophotometer (Thermo Fisher Scientific). The cytokine concentration was determined based on the standard curve, and data analysis was done using Microsoft Excel and GraphPad Prism. All washing steps were performed by washing the plate first three times with 0.05% Tween (Fisher BioReagents, catalog no.: BP337) in PBS and then once with PBS. All reagents were included in the cytokine detection kits unless stated otherwise.

### Western blot

Cells were harvested in cold M-PER lysis buffer (Thermo Fisher Scientific, catalog no.: 78501) or cold in-house lysis buffer (1% Triton X-100, 50 mM Hepes, 10 mM NaCl, 2.5 mM MgCl_2_, 2 mM EDTA, and 10% glycerol) in the presence of phosphatase and protease inhibitors (Thermo Fisher Scientific, catalog no.: A32961 or Thermo Fisher Scientific, catalog no.: A78441). Lysates were analyzed by standard WB protocol using commercial gels (Invitrogen, catalog no.: NW04120) and nitrocellulose membranes (GE Healthcare, catalog no.: 10600004). Primary antibodies used were rabbit anti-phospho-EIF2a (Ser51 phosphorylation, Cell Signaling, catalog no.: 9721S), rabbit anti-GADD34 (Proteintech, catalog no.: 10449-1-AP), and rabbit anti-β-actin (Cell Signaling, catalog no.: 4970S). The detection was done by standard ECL protocol using Goat anti-rabbit horseradish peroxidase (Invitrogen, catalog no.: 31460) secondary antibody and SuperSignal West Pico PLUS Chemiluminescent Substrate (Thermo Fisher Scientific, catalog no.: 34580). Band intensities were quantified in ImageJ Fiji by measuring the signal intensity of each target protein and normalizing it to the corresponding loading control.

### Translation intensity measurement

Puromycin (10 μg/ml; Gibco, catalog no.: A11138-03) was added (or not to nontreated groups) to the DC media for 10 min at 37 °C and 5% CO_2_ before harvesting. Incorporation of puromycin into nascent proteins was assayed by WB as aforementioned using anti–puromycin PE–conjugated antibody (BioLegend, catalog no.: 381503).

## Data availability

Data generated in this study are available upon request from the corresponding author (S.C.F.).

## Supporting information

This article contains supporting information (two figures and one table).

## Conflict of interest

The authors declare that they have no conflicts of interest with the contents of this article.

## Figures and Tables

**Supplemental Figure 2:**