# Supplementary material for: β2-Integrins regulate dendritic cells through nuclear deformation and activation of phospholipase A2 and DNA damage–inducible GADD34
Source: J Biol Chem. 2025 Dec 22;302(2):111093. doi: 10.1016/j.jbc.2025.111093 (PMC12853782; doi:10.1016/j.jbc.2025.111093)
Supplement: Supplemental Table [file mmc1.docx]

| **284 common elements in "Nuclear deformation" and "Integrin deficient"** |
| --- |
| Hdc, Arl5c, Socs3, Sqstm1, Nfkb1, Nfkbib, Dennd5a, Stx11, Saa3, Gadd45b, Ccr7, Nfkbie, Cxcl16, St3gal1, Tma16, Il12b, Ttc39c, Gbp5, Ankrd33b, Mxd1, Flrt3, Cd83, Pxdc1, Gga2, Marcksl1, Traf1, Cyth1, Gpr132, Dusp5, Etv3, Ramp3, Rab11fip1, Rab8b, Stat3, Mx1, Cd40, Map4k4, Scin, Tarm1, Sema7a, Tmem39a, Icam1, Samsn1, Dusp1, Tmtc2, Stat5a, Gls2, Pdzk1ip1, Nr4a2, Csrnp1, Stat4, B3gnt5, Pnrc1, Slc40a1, Tjp2, Rnf19b, Nfkb2, Nr4a3, Cd86, Gpr65, Skil, Havcr2, Pfkfb3, 6530402F18Rik, Peli1, Fas, Ppfibp2, Rel, Osm, Abtb2, Kdm4a, Rnf125, Crem, Pde4b, Ccl5, Ncoa7, Wnt11, Jak2, Slc44a1, Mefv, Ppp1r15a, Mir155hg, Nrg1, Furin, Crebl2, Adora2a, Tbc1d9, Nudt9, Foxp1, H2-Q4, Serpinb9, Cttnbp2nl, H2-Q7, Dgka, Fosb, Myo1g, Carhsp1, Map3k14, Foxo1, Cep126, Pkib, Tes, Nub1, Marcks, Nr4a1, Rcl1, Spic, Slc1a4, Cytip, Filip1l, Fmnl2, Prkar2b, Hemk1, Traf4, Frmd4b, Fyn, S1pr3, Serpinb6b, Gpr85, Arhgap26, Usp22, Arhgap31, Cyth3, Six1, Slamf7, Mmp25, Sdc4, Socs1, Orai2, Tpbg, Il15, Plac8, Slco3a1, Ccdc71l, Calcb, Foxp4, Rnf180, Bcl2l11, Ahcyl2, Relb, Tspan13, Dennd4a, Spag9, Prdm1, Fbxo11, Irs2, Nectin2, Nipal1, Zfp263, Tent5c, Crtc2, 6430571L13Rik, Bcl6, Aff1, Stk40, Pdcd1, Msrb3, Ptprf, Zc3h12c, Pla1a, Rbbp8, Zeb1, Stxbp3, P2ry10, Pde4d, Nabp1, Pgf, Gpcpd1, Il12rb2, Pcgf5, Arl4c, Fnbp1l, Tmem131, Ext1, Cd82, Zfp36, Spsb1, Plat, Ssh1, Klrk1, Pdlim7, Col27a1, Pim2, Cacng8, Il15ra, Klri2, Birc2, Polb, Mllt6, Ocln, Mvp, Cacnb1, Pik3r5, Akap13, Sh3bp4, Zmynd15, Clec2l, Synj1, Atp2b4, Rasd1, Bcl11a, Cyp4a12b, Il1r1, Bco2, H2-Q5, Zbtb10, Gucd1, 4930523C07Rik, Hip1r, Hsf2, Ier5, Clasp2, Kctd14, Cox17, Aebp2, Ppm1k, Cd70, Ly9, Ptpn3, Stk38l, Pld2, Mreg, Shf, Ktn1, Heatr9, Arhgef10l, Gm15663, Mab21l3, Gpbp1, Klri1, Tbc1d15, Carmil1, Vav3, Amigo3, Pdcd1lg2, Tinagl1, Apof, Itga4, Secisbp2l, St6gal1, Rictor, Zfp366, Ccr3, Smurf1, Klrg2, Mfsd6, Creb5, Mier3, Mfsd6l, Bpifc, Syne2, Clec2d, Vcam1, Usp35, Bcl9l, Tet2, Foxq1, Noct, Tal1, Ahnak, Mid1, Adamts7, Mtmr12, Gpr157, Adgrg6, Ssh2, Lancl2, Dlgap3, Grhl1, Chka, Slc6a4, Gm12866, Rab26, Nuak2, Prr7, Rasa2, Timp3, Aldh3a1, Hivep1, Mturn, Ppp1r9a, 1110002J07Rik, AI504432, Fam169b |

**Supplementary table.** List of genes upregulated by nuclear deformation and β2-integrin deficient DCs.
